# High Level Aminoglycoside Resistance and Distribution of Aminoglycoside Resistant Genes among Clinical Isolates of *Enterococcus* Species in Chennai, India

**DOI:** 10.1155/2014/329157

**Published:** 2014-02-04

**Authors:** Elango Padmasini, R. Padmaraj, S. Srivani Ramesh

**Affiliations:** ^1^Department of Microbiology, Dr ALM Post Graduate Institute of Basic Medical Sciences, University of Madras, Chennai 600 113, India; ^2^Department of Paediatric Nephrology, Institute of Child Health and Research Centre, Chennai 600 008, India

## Abstract

Enterococci are nosocomial pathogen with multiple-drug resistance by intrinsic and extrinsic mechanisms. Aminoglycosides along with cell wall inhibitors are given clinically for treating enterococcal infections. 178 enterococcal isolates were analyzed in this study. *E. faecalis* is identified to be the predominant *Enterococcus* species, along with *E. faecium*, *E. avium*, *E. hirae*, *E. durans*, *E. dispar* and *E. gallinarum*. High level aminoglycoside resistance (HLAR) by MIC for gentamicin (GM), streptomycin (SM) and both (GM + SM) antibiotics was found to be 42.7%, 29.8%, and 21.9%, respectively. Detection of aminoglycoside modifying enzyme encoding genes (AME) in enterococci was identified by multiplex PCR for *aac(6′)-Ie-aph(2′′)-Ia*; *aph(2′′)-Ib*; *aph(2′′)-Ic*; *aph(2′′)-Id* and *aph(3′)-IIIa* genes. 38.2% isolates carried *aac(6′)-Ie-aph(2′′)-Ia* gene and 40.4% isolates carried *aph(3′)-IIIa* gene. *aph(2′′)-Ib; aph(2′′)-Ic; aph(2′′)-Id* were not detected among our study isolates. *aac(6′)-Ie-aph(2′′)-Ia* and *aph(3′)-IIIa* genes were also observed in HLAR *E. durans*, *E. avium*, *E. hirae*, and *E. gallinarum* isolates. This indicates that high level aminoglycoside resistance genes are widely disseminated among isolates of enterococci from Chennai.

## 1. Introduction 

Enterococci have emerged as an important multiple-drug resistant nosocomial pathogen reported worldwide. Its resistance to wider range of antimicrobial agents particularly, aminoglycosides, glycopeptides and beta-lactams had increasingly been documented [1]. Although enterococci are intrinsically resistant to low levels of aminoglycosides, high level resistance to aminoglycosides (MIC ≥ 2000 *μ*g/mL) is mediated by acquisition of genes encoding AMEs. High level gentamicin resistance (MIC ≥ 500 *μ*g/mL) in enterococci is predominantly mediated by *aac(6*′*)-Ie-aph(2*′′*)-Ia*, which encodes the bifunctional aminoglycoside modifying enzyme AAC(6′)-APH(2′′). The action of this enzyme in enterococci eliminates the synergistic activity of gentamicin when combined with a cell wall active agent, such as ampicillin or vancomycin. Recently, newer AME genes such as *aph(2*′′*)-Ib*, *aph(2*′′*)-Ic*, and *aph(2*′′*)-Id* have been detected as those conferring gentamicin resistance in enterococci. High level streptomycin and kanamycin resistance in enterococci are mediated by *aph(3*′*)-IIIa* gene encoding aminoglycoside phosphotransferase enzyme, APH(3′)-IIIa [2]. In India, high level aminoglycoside resistance has been reported from different centers; however, studies on prevalence of these resistance genes are limited. The goal of this study is to determine, the rate of high level aminoglycoside resistance and aminoglycoside resistance encoding genes in enterococcal isolates collected from different specimen sources in Chennai, India.

## 2. Materials and Methods

### 2.1. Bacterial Strains

A total of 178 nonidentical clinical isolates of enterococci were obtained from clinical specimens from various tertiary care centers from Chennai, during a period of 2010–2012. Appropriate inpatient details were collected and recorded to avoid identical isolates from the same patient. An Institutional ethical clearance was obtained for conducting this study (reference number: 1168). The strains were initially grown on MacConkey agar (MV082) and *Enterococcus* confirmatory agar (M392) (HiMedia, Mumbai, India). Species characterization was carried out by carbohydrate fermentation test using 1% sugars such as sucrose, sorbose, sorbitol, mannitol, glucose, pyruvate, inulin, ribose, melibiose, raffinose, arabinose, and arginine. All the isolates were confirmed for genus and species by standard protocols [3]. *Enterococcus faecium* and *Enterococcus faecalis* were further confirmed by PCR analysis using specific *ddl*_*E*.*faecium *_ and *ddl*_*E*.*faecalis*_ genes, respectively [4].

### 2.2. Minimum Inhibitory Concentration for Aminoglycosides

The isolates were confirmed as high level aminoglycoside resistant enterococci (HLARE) by considering growth ≥512 *μ*g/mL for gentamicin and ≥2048 *μ*g/mL for streptomycin. The overnight bacterial cultures were adjusted to 0.5 McFarland's turbidity and the inoculum was spot inoculated on the surface of brain heart infusion agar with increasing concentrations of gentamicin and streptomycin antibiotics (HiMedia, Mumbai, India). The plates were incubated at 37°C for 24 hrs and inspected for more than one colony forming units in the spotted area. *Enterococcus faecalis* ATCC 29212 was used as a negative control strain.

### 2.3. Molecular Analysis of Aminoglycoside Modifying Genes by PCR

The primers for AME genes such as *aac(6*′*)-Ie-aph(2*′′*)-Ia*; *aph(2*′′*)-Ib*; *aph(2*′′*)-Ic*; *aph(2*′′*)-Id* and *aph(3*′*)-IIIa* included in this study were previously described [5].

PCR was carried out with reaction tube containing 1 *μ*L template DNA prepared from boiling lysis of bacterial suspension added to a 50 *μ*L reaction mixture containing 25 mM Tris/HCl, 50 mM KCl, 1.5 mM MgCl_2_, 0.2 Mm of each dNTP (Bangalore Genei, India), and 1.5 U *Taq *polymerase (Bangalore Genei, India). First reaction with a pair of 25 pmol each of primers for *aac(6*′*)-Ie-aph(2*′′*)-Ia* and *aph(3*′*)-IIIa *(Sigma Aldrich, USA) and second reaction with *aph(2*′′*)-Ib*, *aph(2*′′*)-Ic*, *aph(2*′′*)-Id* primer sets separately.

Amplification was performed with PCR system (Eppendorf, Germany) and the cycling programs consisted of an initial denaturation (95°C, 5 min) followed by 32 cycles each of denaturation (95°C, 1 min), annealing (58°C, 1 min) and extension (72°C, 1 min), with a final extension of (72°C, 5 min). Each amplification product was resolved by electrophoresis with a 100-base pair molecular weight marker (Real Biotech Corporation, Taiwan) in a 1.2% agarose-Tris-borate-EDTA gel stained with ethidium bromide (0.5 *μ*g/mL) and visualized under gel documentation system (BioRad, USA).  Table 1 shows the product size of all the genes analyzed.

## 3. Results and Discussion

### 3.1. Identification of *Enterococcus* Species

Since early 1970s, Enterococci were considered as nosocomial pathogens. The incidences of high level GM/SM resistance have been disseminated in many *Enterococcus* species. Since then, the high level aminoglycoside resistance has become a serious problem in most of the health care settings; identification of clinical isolates of enterococci up to species level is essential for an appropriate management of the infection. The predominant species observed in our study was *E. faecalis* 86/178 (48.3%) as observed in previous studies [6] in our region. Other than *E. faecium *which was 80/178 (44.9%), we have also obtained *E. avium* (2%), *E. hirae* (1.6%), *E. durans* (0.6%), *E. gallinarum,* and *E. dispar *(1%). The species distribution and specimen source of isolates were listed in Table 2.

### 3.2. High Level Aminoglycoside Resistance in Enterococcal Isolates

Aminoglycoside antibiotics are considered efficient in treating serious infections caused by both gram positive and gram negative organisms. Due to acquisition of extrinsic resistance to high level aminoglycoside antibiotics in enterococci, these strains gain importance in clinical settings. A total of 178 enterococcal isolates were screened by MIC method, 76/178 (42.7%) were HLGR (MIC ≥ 512 *μ*g/mL) for gentamicin and 53/178 (29.8%) were HLSR (MIC ≥ 2048 *μ*g/mL) for streptomycin (Table 3). Although the clinical use of streptomycin for enterococci has long been restricted due to intrinsic low level resistance (LLR), the present study revealed HLSR strains.

A total of 129/178 (72.47%) high level aminoglycoside resistant enterococci (HLGR and HLSR) were observed among our study isolates.

Recent studies also indicated HLGR to be more common in all species of enterococci than HLSR. Similarly, we had observed HLGR to be more predominant than HLSR in our study isolates. One *E. avium*, *E. hirae, E. durans,* and *E. gallinarum* isolates were exhibiting MIC of ≥512 *μ*g/mL for gentamicin and ≥2048 *μ*g/mL for streptomycin antibiotics. The reports on resistance carried by species other than *E. faecalis* and *E. faecium* were observed from late 1990s [8]. A surveillance study that analyzed 20 European countries had reported 32% and 22% HLGR and 41% and 49% HLSR among gentamicin resistant *E. faecalis* and* E. faecium*, respectively [9]. A very recent study conducted in Iran [10] had reported around 60.45% HLGR strains in their region. This is higher than our present report. They were suggesting cotransfer of these resistance genes along with VRE for the higher percentage of HLGR in their study. However, studies on AME gene profile were not done frequently in our region.

### 3.3. PCR Identification of HLAR Genes in Enterococci

**Figure 1:**
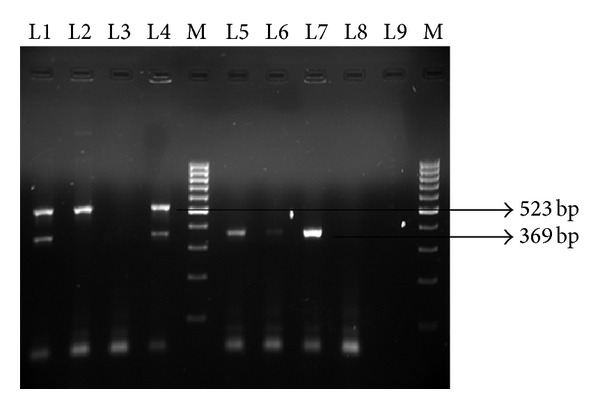
Amplified products of AME genes generated by multiplex PCR. L1, L2, L4-*aph(3*′*)-IIIa* positive (523 bp); L1, L4, L5, L6, L7 *aac(6*′*)-Ie-aph(2*′′*)-Ia* positive (369 bp); M-marker (100 bp DNA ladder).

All 178 enterococcal isolates were analyzed for the presence of aminoglycoside modifying enzyme coding genes (Figure 1).

High level gentamicin resistance is primarily due to the presence of bifunctional enzyme *aac(6*′*)-Ie-aph(2*′′*)-Ia* which also confers high level resistance to amikacin, tobramycin, kanamycin, netilmicin, and dibekacin except streptomycin [11]. *aph(2*′′*)-Ib* was first detected from *E. faecium* and *E. coli* and confers high level resistance to gentamicin, tobramycin, amikacin, kanamycin, netilmicin, and dibekacin but not to amikacin. *aph(2*′′*)-Ic* confers HLR to gentamicin, tobramycin, and kanamycin while the strains carrying them can be treated with amikacin, netilmicin, and streptomycin in combination with cell wall inhibitors. Earlier this gene was shown to be present in *E. gallinarum*; but it had also been reported in isolates obtained from farm animals and in *E. faecalis* and *E. faecium* [12]. *aph(2*′′*)-Id* was reported in *E. casseliflavus* and has similar mechanism to that of *aph(2*′′*)-Ib* [13].

*aac(6*′*)-Ie-aph(2*′′*)-Ia* gene was found in 38.2% of enterococcal isolates in our study. But, out of 76 strains of HLGR identified by MIC method, only 52 strains (68.4%) carried *aac(6*′*)-Ie-aph(2*′′*)-Ia* gene. However, 24/76 (31.57%) isolates that were high level gentamicin resistant and 12/53 (22.64%) isolates that were high level streptomycin resistant did not carry any of the genes tested. In a previous study [14], all the high level gentamicin resistant *E. faecalis* and *E. faecium* isolates were found to carry *aac(6*′*)-Ie-aph(2*′′*)-Ia* gene.

Newer aminoglycoside resistance genes such as *aph(2*′′*)-Ib*, *aph(2*′′*)-Ic,* and *aph(2*′′*)-Id* also found to encode high level resistance to gentamicin (>500 *μ*g/mL) were not detected among our study isolates.

Another most important gene tested in our study, *aph(3*′*)-IIIa* (GenBank Accession number: KF550184) was detected among 40.4% (72/178) isolates of enterococci. Out of 53 high level streptomycin resistant isolates, 41 (77.4%) carried *aph(3*′*)-IIIa* gene.

20.2% (39/178) of strains carried both *aac(6*′*)-Ie-aph(2*′′*)-Ia* and *aph(3*′*)-IIIa* genes in our study. Out of 39 (21.9%) HLAR isolates which were resistant to both gentamicin (MIC > 512 *μ*g/mL) and streptomycin (MIC > 2048 *μ*g/mL) by MIC, 19 (48.7%) isolates carried both the genes, and the remaining 38.5% isolates had one of the genes either *aac(6*′*)-Ie-aph(2*′*)-Ia* or *aph(3*′*)-IIIa* gene, while 12.8% isolates did not carry neither of the genes tested.

*E. faecalis*, the predominant isolate of our study was found to carry *aac(6*′*)-Ie-aph(2*′′*)-Ia* gene in 34/86 (39.5%) isolates and *aph(3*′*)-IIIa* gene in 34/86 (39.5%) isolates. Another predominant pathogenic species obtained among our study isolates was *Enterococcus faecium*. 30/80 (37.5%) *E. faecium* isolates with an MIC range between 128 *μ*g/mL and 512 *μ*g/mL carried the bifunctional enzyme coding gene *aac(6*′*)-Ie-aph(2*′′*)-Ia*.

Each of the HLAR *E. avium*, *E. hirae*, and *E. durans* had both the genes while another *E. avium* strain carried *aph(3*′*)-IIIa* with streptomycin MIC of 1024 *μ*g/mL. One of the two *E. gallinarum* strains isolated was HLAR and it carried both aminoglycoside resistance genes *aac(6*′*)-Ie-aph(2*′′*)-Ia *and *aph(3*′*)-IIIa*, while the other strain carried *aph(3*′*)-IIIa* gene alone but with an MIC of ≥16 *μ*g/mL for gentamicin and >64 *μ*g/mL for streptomycin. One of the two *E. dispar* isolates in our study was found to be HLGR + HLSR and carried *aph(3*′*)-IIIa* gene (see Table 4).

## 4. Conclusion

In our study, we had observed enterococcal isolates with phenotypic resistance towards high level gentamicin and streptomycin antibiotics without presence of respective AME gene. This may be due to the expression of genes other than genes analyzed in this study. The coexistence of *aac(6*′*)-Ie-aph(2*′′*)-Ia* and *aph(3*′*)-IIIa* was observed in 20.2% of the isolates.

Though an array of AMEs are responsible for HLAR status among *Enterococcus* species, we have demonstrated *aac(6*′*)-Ie-aph(2*′′*)-Ia* and *aph(3*′*)-IIIa* genes more frequently occurring than other genes. This observation emphasizes the restricted gene distribution and transfer of resistant genes within a geographical region. Hence, surveillance studies should be conducted among *Enterococcus* isolates from different sources in any given geographical area to document the AME gene profile. Our study is the first to report resistance gene analysis among the *Enterococcus* species in India.

## Figures and Tables

**Table 1:** Primers and their sequences for aminoglycoside resistance encoding genes used in multiplex PCR.

Genes	Primer sequences (5′-3′)		Size of PCR product (bp)
*aac(6′)-Ie-aph(2′′* *)-Ia *	F: CAGGAATTTATCGAAAATGGTAGAAAAG	R: CACAATCGACTAAAGAGTACCAATC	369
*aph(2′′)-Ib *	F: CTTGGACGCTGAGATATATGAGCAC	R: GTTTGTAGCAATTCAGAAACACCCTT	867
*aph(2′′)-Ic *	F: CCACAATGATAATGACTCAGTTCCC	R: CCACAGCTTCCGATAGCAAGAG	444
*aph(2′′)-Id *	F: GTGGTTTTTACAGGAATGCCATC	R: CCCTCTTCATACCAATCCATATAACC	641
*aph(3′)-IIIa *	F: GGCTAAAATGAGAATATCACCGG	R: CTTTAAAAAATCATACAGCTCGCG	523

**Table 2:** Distribution of *Enterococcus *species from various clinical specimens.

Source of the isolates	Distribution of *Enterococcus* spp. (*n* = 178)							Total
	*E. faecalis *	*E. faecium *	*E. durans *	*E. avium *	*E. hirae *	*E. dispar *	*E. gallinarum *	
Urine	62	38	—	2	3	2	—	107
DFU^†^	9	2	1	1	—	—	—	13
Blood	7	35	—	—	—	—	2	44
Pus	5	3	—	1	—	—	—	9
CSF	—	1	—	—	—	—	—	1
Vaginal/semen swab	3	1	—	—	—	—	—	4

Total	86	80	1	4	3	2	2	178

^†^DFU: diabetic foot ulcer isolates.

**Table 3:** Results of minimum inhibitory concentration of enterococcal isolates to gentamicin and streptomycin (*n* = 178).

MIC (*μ*g/mL)	Gentamicin (*n* = 178)	Streptomycin (*n* = 178)
0.50	0	0
1	0	0
2	1	1
4	6	0
8	8	0
16	44	0
32	15	0
64	5	12
128	19	85
256	4	11
>512	76	2
1024	ND^†^	14
>2048	ND^†^	53

^†^ND: not done; HLGR > 500 *μ*g/mL; HLSR > 2000 *μ*g/mL [7].

**Table 4:** Results of high level aminoglycoside resistance and distribution of aminoglycoside modifying enzyme encoding genes among *Enterococcus* spp.

HLARE (MIC and detection of genes by PCR)	Distribution of high level aminoglycoside resistance in *Enterococcus* spp. (*n* = 178)							Total
	*E. faecalis * (86)	*E. faecium* (80)	*E. durans * (1)	*E. avium* (4)	*E. hirae* (3)	*E. dispar* (2)	*E. gallinarum* (2)	
HLGR*	32	39	1	1	1	1	1	76
HLSR*	21	27	1	1	1	1	1	53
*aac(6′)-Ie-aph(2′′* *)-Ia *	34	30	1	1	1	—	1	68
*aph(2′′)-Ib *	—	—	—	—	—	—	—	—
*aph(2′′)-Ic *	—	—	—	—	—	—	—	—
*aph(2′′)-Id *	—	—	—	—	—	—	—	—
*aph(3′)-IIIa *	34	30	1	2	1	2	2	72

*HLGR; HLSR; MIC screening.
